# Mycobacterial glycoproteins: a novel subset of vaccine candidates

**DOI:** 10.3389/fcimb.2014.00133

**Published:** 2014-09-17

**Authors:** Antonio Facciuolo, Lucy M. Mutharia

**Affiliations:** Department of Molecular and Cellular Biology, University of GuelphGuelph, ON, Canada

**Keywords:** *Mycobacterium*, glycoproteins, paratuberculosis, bovine tuberculosis, vaccination

Over the last two decades significant research efforts and resources have been devoted to identifying mycobacterial proteins of value to diagnostic assays and vaccine formulations. These scientific endeavors were often preceded by first identifying a target population of proteins, for example cytoplasmic, cell envelope, or extracellular. However, many of these endeavors have overlooked the posttranslational modifications (PTM) to which these protein subsets may be targets of. Consequently, we may be missing essential molecular information relevant to the function and antigenicity of these effector molecules, and in turn the pathobiology of these bugs. Moreover, heterologous expression of mycobacterial proteins in hosts lacking homologous PTM systems negates any functional and/or antigenic role the PTM may impart. Functional and pathogenic roles of PTMs, such as protein glycosylation, have been reported for other Gram-positive bacteria, especially in reference to mucosal pathogens (reviewed by Szymanski and Wren, 2005). Our objective in this piece is to shed light on the lack of PTM studies so that current and future researchers hunting for diagnostic and vaccine candidates shall: (i) be made aware of PTMs of mycobacterial proteins, and (ii) come to understand their growing importance to both pathobiology and immunogenicity. Interestingly, the last 5 years has only yielded 2 reports on the global analyses of mycobacterial glycoproteins, both for *M. tuberculosis* (Mtb), and over the last decade only a few studies demonstrated the importance of PTMs to the pathobiology and antigenicity of select proteins in Mtb, *M. bovis*, and *Mycobacterium avium* subsp. *paratuberculosis* (MAP). In this communication we present the opinion that in a world of cross-reactive epitopes, and high amino acid sequence similarity between *M. bovis*, MAP, and their saprophytic counterparts, PTM diversity amongst these species may confer a new level of epitope-specificity for select antigens. We will also highlight that in the case of protein glycosylation, the type and extent of glycosyl moieties should not been seen as having the same outcome. Furthermore, the presence or absence of the PTM of proteins included in subunit vaccine formulations or attenuated strains, may enhance or mask the processing, presentation, and immunogenicity of relevant epitopes. Collectively, these data call to action critical analyses of the components we use to formulate mycobacterial vaccines. As the vast majority of PTM studies have focused on glycosylation, this PTM will be the focal point of discussion.

## History of mycobacterial glycoproteins

Espitia and Mancilla (1989) first identified 3 glycoproteins in Mtb H37Rv culture filtrate (CF) using mannose-binding lectin Concanavalin A. Immunoblotting revealed 2 of the glycoproteins (Rv1860-Apa, Rv0934) were reactive with 38% of serum samples from active TB patients tested; no reactivity was detected with healthy control samples. Subsequent studies mapped the glycosylated peptides of the Mtb Apa adhesin protein using endopeptidase digestion and MS analysis (Dobos et al., 1995). Horn et al. (1999) examined the antigenicity of the Apa protein in *M. bovis* BCG-immunized guinea pigs. Using MS, they demonstrated mannosylation of native Apa purified from Mtb and *M.bovis*, mannosylation of recombinantly expressed Apa in *M.smegmatis*, however with 2–3 additional mannose residues per branch, and the lack of mannosylation of recombinant *E.coli* Apa. In T-cell assays, native Mtb and *M. bovis* Apa protein elicited similar levels of lymphoproliferation, whereas recombinant *E.coli* Apa showed negligible activity. Interestingly, recombinant *M. smegmatis* Apa lymphoproliferation was 10-fold less despite similar, albeit increased, glycosylation patterns as the native proteins. This report was the first to demonstrate that mycobacterial proteins stimulate more robust immunological responses as glycoconjugates, and that species-specific differences in glycosylation alter this response. Interestingly, glycosylation differences for Apa of Mtb and *M.marinum* have also been demonstrated, however, the functional implication of this is yet to be elucidated (Coddeville et al., 2012). To date, no follow up studies have addressed, (i) differential mannosylation in *M. smegmatis*, *M. bovis*, and Mtb, and (ii) why variation in glycosylation results in an altered immunological response.

Recently two complimentary approaches, Con A lectin affinity chromatography, and MS analysis for O-linked hexosylation, were taken to address global protein glycosylation in Mtb CF. Interestingly, the MS analyses identified 13 glycoproteins in Mtb CF in contrast to the 41 captured by affinity chromatography (González-Zamorano et al., 2009; Smith et al., 2014). Liu et al. (2013) first linked a functional and virulent role to the Mtb mannosyl transferase membrane protein (Rv1002c) by establishing a Δ*Rv1002c* mutant. Murine alveolar macrophages co-cultured with the ΔRv1002c mutants showed a 50% reduction in uptake over H37Rv. Moreover, median survival of SCID mice challenged with the Δ*Rv1002c* mutants increased by 41 days over H37Rv. Collectively, these data highlight the significant contribution of glycoproteins to infectivity and virulence of Mtb. In another study, MS mapping of the *M.bovis* glycoprotein MPB83 identified a unique mannose linkage not previously reported in Mtb glycoproteins (Michell et al., 2003). These data raise interesting questions such as: (i) how this linkage may affect interactions with immunological receptors such as the TCR, MHC, and/or BCR by enhancing/masking/modulating its binding, and (ii) whether these linkages are part of the antigenic determinant, and if so, do they impart species-specificity even in highly homologous proteins.

## Glycosylation and antigenicity

Glycosylation-dependent peptide epitopes were demonstrated by ELISA using mannosidase-treated recombinant Mtb Apa expressed in *Streptomyces* (Lara et al., 2004). Reactivity of serum from TB patients was significantly reduced after Apa was treated with mannosidase. In our lab, similar observations were found with 17 MAP CF glycoproteins purified by Con A lectin-affinity chromatography. Both bovine and ovine paratuberculosis serum contained antibodies against these antigens. However, only bovine serum activity was abrogated after mannosidase treatment of these antigens (Mutharia et al., 1997). Currently, our lab is identifying these, and other MAP glycoproteins, among the complement of secreted proteins we recently identified (Facciuolo et al., 2013). Using Con A lectin blotting on MAP CF proteins concentrated and resolved by RPLC we can reproducibly detect 14 reactive bands by SDS-PAGE in the range of 10–75 kDa (Facciuolo and Mutharia, unpublished). Applying Con A lectin-affinity chromatography 9 unique bands were eluted, and resolved by SDS-PAGE. By 2D PAGE, 6 of these 9 unique bands have horizontal spot series (on average 3 spots) indicative of PTMs. To our knowledge, this is the most extensive reporting on MAP glycoproteins.

Recently, protein glycosylation on T-cell antigenicity and vaccination was elucidated using native and recombinant Apa-protein isoforms of Mtb (Nandakumar et al., 2013). In BCG+ PPD+ individuals, CD4+ cytokine-secreting cells were significantly higher in PBMCs stimulated with the native protein than the recombinant isoform. Moreover, the native glycosylated Apa uptake was higher than that of the unglycosylated recombinant isoform in dendritic cells from PBMCs of both BCG+ and BCG− individuals. *In vitro* analyses showed only the glycosylated isoform bound recombinant human innate immune receptors such MR, DC-SIGN, and DC-SIGNR. Despite these significant differences, both protein isoforms were equally protective in BALB/c mice as subunit vaccines stimulating similar CD4+ cytokine and serum antibodies responses. These data suggest that glycosylation is necessary for antigenicity, but not induction of protective immunity. The outcomes of these data are quite interesting when also considering the earlier studies by Ishioka et al. (1992). Using synthetic peptides, three GlcNAc-position-dependent outcomes were observed in BALB/c immunized mice. Positioning of the GlcNAc conjugated amino acid could either limit lymphoproliferative responses to the glycosylated peptide only, abolish antigenicity of the peptide, or result in antigenicity of both the glycosylated and unglycosylated peptide isoforms. The authors speculated that the consequences were the result of MHC-TCR interactions where the glycoconjugate: is the antigenic determinant, interfered with the MHC binding site, or was distal of the critical MHC binding pocket, respectively. In light of these data, careful analyses and consideration of both glycosylation status and their site of attachment on proteins are needed to ensure the efficacy of these molecules in stimulating the desired immunological response. These considerations should also be applied in the development of attenuated strains as the presence or absence of glycosylated proteins may either enhance/mask protective stimulators/inducers of innate/adaptive immunity.

The complexity of glycosylation in addition to the consequences it carries calls to action a bottom-up approach in screening for novel vaccine candidates. The potential of these effector molecules to contain species-specific glycosylation markers may benefit vaccine formulations, in addition to diagnostic assays. The technical hurdles in obtaining the necessary quantity and fidelity of glycoproteins from slower growing *Mycobacterium* currently presents the greatest challenge in this field, but has had some measure of success via heterologous expression, or native purification by lectin-affinity purification coupled with LC. These technical challenges might best be served by efforts to glycoengineer PTM systems in faster growing species. The sum of these data may aid in identifying those effector molecules, (i) that exclusively require their PTM for antigenicity, (ii) where the PTM is dispensable for protective immunity, and (iii) containing new subsets of protective or relevant antigens that had been masked by the presence of a PTM.

### Conflict of interest statement

The authors declare that the research was conducted in the absence of any commercial or financial relationships that could be construed as a potential conflict of interest.

## Abbreviations

LC: liquid chromatography
MS: mass spectrometry
MHC: major histocompatibility complex
TCR: T-cell receptor
BCR: B-cell receptor
BCG: Bacillus Calmette-Guérin
PPD: purified protein derivative
PBMC: peripheral blood mononuclear cell
MR: mannose receptor
DC-SIGN: dendritic cell-specific intercellular adhesion molecule-3-grabbing non-integrin.
